# Epigenetic age acceleration as a biomarker of amyotrophic lateral sclerosis severity?

**DOI:** 10.1016/j.ebiom.2024.105470

**Published:** 2024-11-23

**Authors:** Sebastian A. Lewandowski, Lara Kular, Maja Jagodic

**Affiliations:** Department of Clinical Neuroscience, Karolinska Institutet, Center for Molecular Medicine, Karolinska University Hospital, Stockholm, Sweden

Amyotrophic lateral sclerosis (ALS) is a devastating neurodegenerative disease characterised by the progressive loss of motor neurons leading to death, most commonly due to neuromuscular respiratory failure. The causes of ALS are multifactorial, with clear contribution from genetic factors to both familial and sporadic forms, as well as increasing evidence implicating environmental factors in ALS risk and progression.^1^ A substantial heterogeneity regarding age at onset and unpredictable survival rate supports a complex interplay between multiple risk factors, superimposed by ageing processes.

In the November 2024 issue of *eBioMedicine*, Zhao and colleagues aimed to connect ageing as a risk factor together with the epidemiological risk factors and exposures.^2^ They provide first reported evidence that epigenetic age acceleration (AccelGrimAge), measured in the peripheral blood from patients with ALS, associates with life expectancy in males and occurs in people with occupations typically associated with elevated risk for ALS. Their well-designed and data-rich ALS cohort allows for robust inference of epigenetic age acceleration and solid modelling of association with a range of clinical parameters, i.e., onset type, sex, ALS Functional Rating Scale-Revised (ALSFRS-R), as well as occupational types and associated exposures.

The functioning of the human body gradually declines; however, this decline assumes a different pace in different individuals and often varies across tissues and cell types within the same individual. Epigenetic clocks exploit DNA methylation levels on a set of CpG sites to infer the biological age of an individual, tissue, or cell type, which in turn reflects their functional capacity better than the individual's chronological age. Numerous epigenetic clocks have been developed, shifting from the concept of predicting chronological age to predicting the risk of functional age-related outcomes. Zhao and colleagues utilised GrimAge, a second-generation epigenetic clock developed using algorithms trained to find methylation signals associated with chronological age, sex, smoking and the levels of seven plasma proteins.^3^ As such, GrimAge correlates with many lifestyle factors and exposures and is a strong predictor of time to age-related diseases and mortality.^4^ Zhao and colleagues adjusted GrimAge for chronological age to measure epigenetic age acceleration and further corroborated the associations using another epigenetic clock, DunedinPoAM, trained on the pace of biological ageing.^5^ Although the mechanistic underpinnings of epigenetic clocks remain unknown, they have biomarker potential for cognitive functions and for a wide range of neurodevelopmental, neuropsychiatric, and neurodegenerative disorders^6^^,^^7^ and hold promise to inform clinical decisions in the future.

Beside clinical predictors, blood protein biomarkers such as neurofilament light chain (NfL) are now considered to be integrated to the design of ALS trials as prognostic biomarkers.^8^ Although advanced chronological age is considered a prognostic factor of poor outcome, epigenetic clocks and epigenetic age acceleration may prove more advantageous compared to protein markers both conceptually and methodologically. Epigenetic clocks represent a compendium of factors and processes leading to functional age acceleration, with recent studies suggesting that they are comprised of different modules driven by distinct biological processes relevant for inferring the body's functional capacity.^9^ Practically, DNA methylation levels are assayed from the same type of biosample used for measurements of genetic or protein markers, while being less susceptible to sample handling and more stable than protein markers such as NfL.

The study brings forth an unanswered question of causality: is epigenetic age acceleration primary or secondary to ALS neurodegeneration? For example, does the epigenetic clock accelerate due to occupational exposures (specifically captured by the clock) leading to the increased risk of developing ALS, or alternatively, is the accelerated epigenetic age a manifestation of the neurodegenerative process independent of the occupational choice? Both interpretations appear non-mutually exclusive and therefore plausible. Occupational factors likely precede the asymptomatic mechanisms of ALS neurodegeneration, which favours the primary causal interpretation. However, epigenetic acceleration in peripheral blood cells may also be a secondary consequence of established pathology. Accordingly, one can speculate that blood epigenetic ageing can reflect similar mechanisms occurring in cells of the central nervous system, which would circumvent the challenge of assessing cellular functional decline in the primary affected organ.^6^^,^^7^^,^^10^ A study of epigenetic clocks in a wider population and across multiple relevant tissues could answer this question, making further research warranted and necessary.

The study by Zhao and colleagues provides a mechanistic convergence of two previously separated epidemiological concepts and demonstrates that accelerated ageing is associated with ALS severity and occupational ALS risks. These findings may open new avenues in the clinical care of patients with ALS and potentially other neurodegenerative diseases, where epigenetic ageing in the blood might assist in informing clinical decisions. The need for biomarker-guided patient stratification becomes more evident in light of the recent shift towards precision medicine in ALS, stipulating biomarker-guided administration of advanced gene therapy modalities in distinct groups of patients.

## Contributors

Conceptualisation: S.A.L, L.K. and M.J.; Literature search: S.A.L., L.K. and M.J.; Drafting the manuscript: S.A.L. and M.J., Reviewing/editing manuscript: S.A.L., L.K. and M.J. All authors read and approved the final version of the manuscript.

## Declaration of interests

S.A.L. received a lecture honorarium from Columbia University, NY, USA and travel support for attending the MND Conference 2024 as a plenary speaker. L.K. and M.J. declare no competing interests.
